# Mental health risks in pregnancy and early parenthood among male and female parents following unintended pregnancy or fertility treatment: a cross-sectional observational study

**DOI:** 10.1186/s12884-024-07082-x

**Published:** 2024-12-26

**Authors:** Naoki Mizunuma, Keiko Yamada, Takashi Kimura, Yutaka Ueda, Takashi Takeda, Takahiro Tabuchi, Kunihiko Kurosaki

**Affiliations:** 1 Department of Legal Medicine, Toho University Graduate School of Medicine, 5-21-16 Omori-nishi, Ota-ku, Tokyo, 143-8540 Japan; 2 Tokyo Kagurazaka Law Office, Tokyo, Japan; 3 Division of Forensic Medicine, Department of Social Medicine, Faculty of Medicine, Tottori University, Yonago, Tottori Japan; 4 Pain Medicine, Juntendo University Graduate School of Medicine, Tokyo, Japan; 5 Department of Public Health, Hokkaido University Graduate School of Medicine, Sapporo, Hokkaido Japan; 6 Department of Obstetrics and Gynecology, Osaka University Graduate School of Medicine, Suita, Osaka Japan; 7 Division of Women’s Health, Research Institute of Traditional Asian Medicine, Kindai University, Osaka-Sayama, Osaka Japan; 8 Division of Epidemiology, School of Public Health, Tohoku University Graduate School of Medicine, Sendai, Miyagi Japan; 9 Cancer Control Center, Osaka International Cancer Institute, Osaka, Japan; 10 Department of Legal Medicine, Toho University School of Medicine, Tokyo, Japan

**Keywords:** Mental health, Pregnancy, Parenthood, Unintended pregnancy, Fertility treatment

## Abstract

**Background:**

Unintended pregnancy at higher risk of perinatal mood disorders; however, concurrent factors such as socioeconomic conditions may be more critical to mental health than pregnancy intention. Mental health risks among individuals undergoing fertility treatment are inconsistent. We investigated mental health risks during pregnancy and parenthood in parents who conceived unintentionally or through fertility treatment compared to those who conceived naturally and intentionally.

**Methods:**

We conducted a web-based study with 10,000 adults ≥ 18 years old, either pregnant or with a child aged < 2 years. Male and female respondents weren’t couples. We analyzed 1711 men and 7265 women, after filtering out invalid responses. We used a questionnaire including conception methods (e.g., naturally conceived intended/unintended pregnancies, fertility treatment such as scheduled intercourse or ovulation inducers [SI/OI], intrauterine insemination [IUI], and in-vitro fertilization or intracytoplasmic sperm injection [IVF/ICSI]) and mental health risks (e.g., psychological distress, chronic pain, death fantasies). Using a modified Poisson regression, we estimated relative risks (RR [CI]) for mental health risks compared to those with intended pregnancies.

**Results:**

Unintended pregnancy showed higher mental health risks during pregnancy in both genders, with women having significantly higher psychological distress, chronic pain, and death fantasies (RR 1.63 [1.05–2.54], RR 1.63 [1.14–2.33], and RR 2.18 [1.50–3.18], respectively). Women’s death fantasies risk remained high in parenthood: RR 1.40 (1.17–1.67). In relation to fertility treatments, men using SI/OI during their partner’s pregnancy showed higher mental health risks, especially for chronic pain (RR 1.75 [1.01–3.05]). Men who underwent IUI showed higher mental health risks during parenthood, notably death fantasies (RR 2.41 [1.13–5.17]). Pregnant women using SI/OI experienced higher mental health risks, with a significant risk of chronic pain (RR 1.63 [1.14–2.33]). Pregnant women using IVF/ICSI had a significantly lower risk of chronic pain (RR 0.44 [0.22–0.87]), but women who used IVF/ICSI had a significantly higher risk of death fantasies during parenthood (RR 1.40 [1.04–1.88]).

**Conclusions:**

Mental health risks vary by parenting stage (pregnancy or early parenthood) and gender, especially for those who conceived unintentionally or through fertility treatment. Both stages require adaptable mental health support for all parents.

**Trial registration:**

N/A (non-interventional study).

**Supplementary Information:**

The online version contains supplementary material available at 10.1186/s12884-024-07082-x.

## Background

There are several methods of intentional and assisted conception and all have unique perinatal and postnatal mental health risks. Pregnancy intentions often influence parental psychological factors during pregnancy and post-delivery (e.g., unintended pregnancies can cause unhappiness) [1]. Since 1978, in vitro fertilization (IVF) has provided an option for infertility problems. However, couples undergoing fertility treatment may experience substantial physical, psychological, and financial burdens, including clinic visits, treatment costs, and planning intimacy around menstrual cycles [2, 3]. Infertile couples may have a higher risk of psychological disorders [4]. Individuals undergoing fertility treatment may experience psychological distress, including depression [5], anxiety [6], anger, frustration, isolation, and death fantasies [7].

Such negative psychosocial factors can trigger somatic symptoms like chronic pain, which can be affected by physiological, psychological, and social factors [8]. Chronic pain during pregnancy and postpartum is a common issue, with studies showing that up to 70% of pregnant women experience low back pain and 45% report pelvic pain [9]. Male partners may also experience chronic pain, such as back pain or headaches [10]. Furthermore, there is a bidirectional relationship between chronic pain and mental health issues such as depression and anxiety [11]. For instance, parents experiencing chronic pain during pregnancy are at a higher risk of developing postpartum depression [12]. Conversely, psychological distress such as anxiety and depression during pregnancy is associated with an increased risk of developing chronic pain after childbirth [13]. Given these interrelationships, it is important to consider chronic pain alongside mental health outcomes when examining the effects of different conception methods.

Couples must manage these burdens until the birth of their child, and some cease fertility treatment owing to psychological stress [6]. Parents also face post-pregnancy challenges. Mental health problems (e.g., depression or anxiety, post-traumatic stress disorder) may arise during pregnancy and post-delivery [14]. Psychological stress in either parent during pregnancy may engender bonding disorders [15]. Furthermore, poor perinatal and postnatal mental health in both men and women are risk factors for child abuse or maltreatment [16].

We chose to investigate both unintended pregnancies and pregnancies resulting from fertility treatments in this study because, despite their differences, they represent opposite ends of the “pregnancy intentionality spectrum” and both have the potential to significantly impact mental health. By examining both types of pregnancies, we aim to explore how different circumstances surrounding pregnancy intention can lead to varying mental health outcomes. Comparing these two types of pregnancies allows us to identify different mechanisms that might influence mental health in these distinct contexts.

Several studies have reported associations between conception methods and female perinatal mental health. Women who have experienced naturally conceived unintended pregnancies have a higher risk of psychological distress [17] and maternal depression [18], particularly during postpartum [19, 20], than women with naturally conceived intended pregnancy. In contrast, the postpartum mental health risks for women who have undergone fertility treatment, compared with those who have conceived naturally, vary depending on individual circumstances and the type of treatment received. While some studies suggest that women who have conceived through fertility treatment are not at higher risk of postpartum depression or anxiety [21], other research suggests a higher prevalence of mental health problems, such as depression, obsessive-compulsive symptoms, and somatization, in these women [22, 23]. In addition, unintended pregnancy has been associated with an increased risk of mental health problems in men, particularly depression. A systematic review and meta-analysis found that men who became unintended fathers were more than twice as likely to report mental health problems, including depression, compared with men who had planned pregnancies [24]. Similarly, men who experience fertility problems may report lower sexual and personal quality of life, leading to social distancing and increased psychological distress [25–27]. In contrast, few studies have assessed the specific mental health outcomes of women facing fertility problems, although some evidence suggests that these women may also face increased emotional distress during the perinatal period [23]. Thus, both unintended pregnancy and fertility problems may have significant psychological effects on both mothers and fathers, although the exact nature and extent of these effects may vary.

We hypothesized that understanding the distinct mental health risks associated with naturally conceived unintended pregnancies and those resulting from fertility treatments could provide a more comprehensive understanding of how pregnancy intention affects mental health. To test this, we investigated the association between conception methods (i.e., naturally conceived intended pregnancy, naturally conceived unintended pregnancy, pregnancy through fertility treatment) and mental health risks (psychological distress, prevalence of chronic pain, death fantasies) among male and female parents who were either pregnant or had children aged < 2 years.

## Methods

### Study design and population

**Fig. 1:**
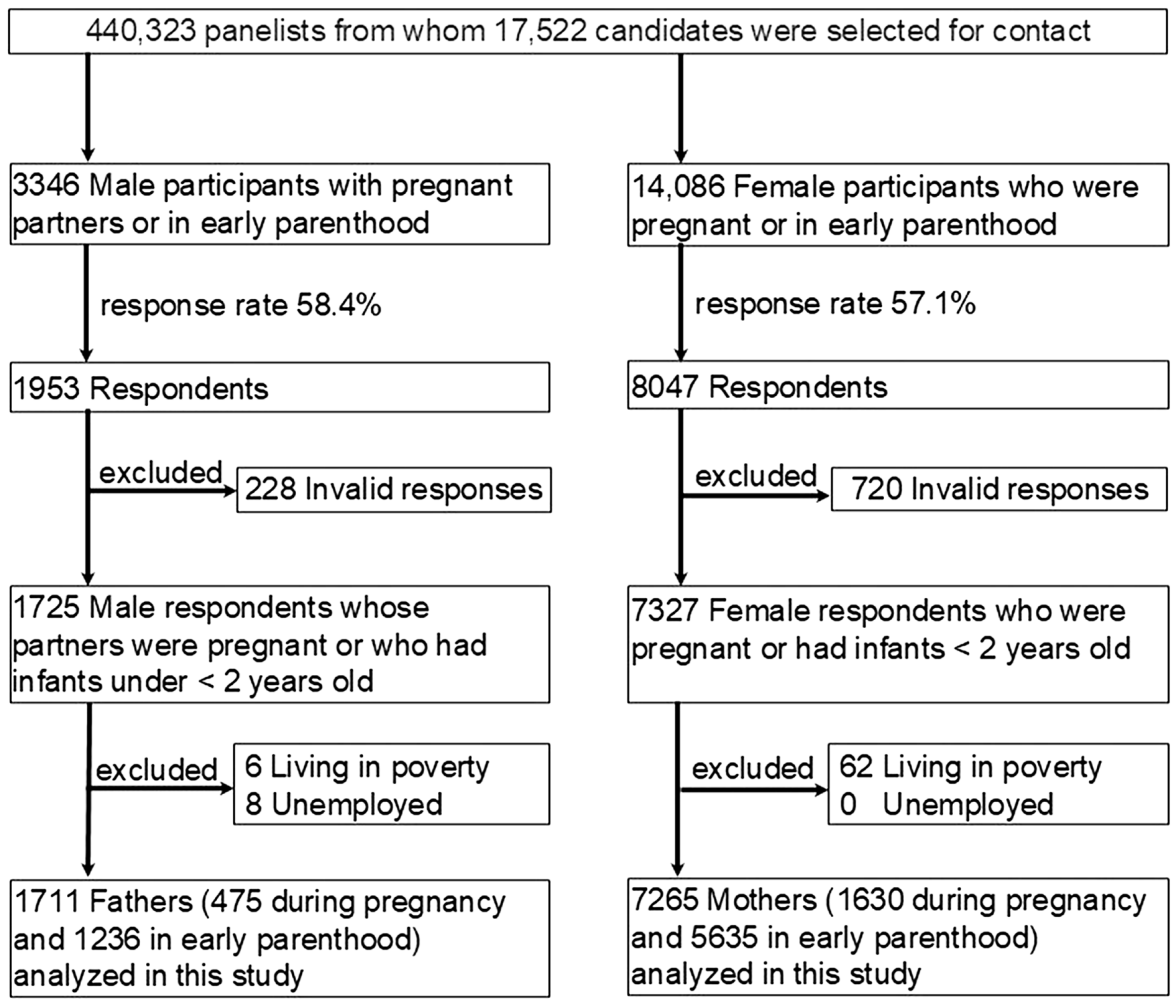
Enrollment process. Parents aged ≥ 18 years, either expecting a child or with a child aged < 2 years, were recruited from 28 July to 30 August 2021

We used data from a cross-sectional web-based special survey conducted in 2021 as part of Japan Coronavirus Disease (COVID)-19 and Society Internet Survey (JACSIS) related to perinatal and early parenthood. This survey specifically targeted pregnant women and parents with children under two years old. It is important to note that the participants in this survey are independent and do not overlap with those in the main JACSIS survey, which covers a broader demographic. The data for this study were exclusively drawn from this non-overlapping subset of the JACSIS panel. Figure 1 shows the participant enrollment process. Random sampling using a computer algorithm was used to recruit participants. All participants electronically provided informed consent before responding to the questionnaire. A total of 440,323 panelists registered with a Japanese internet survey agency, Rakuten Insight, Inc. (based in Tokyo, Japan). A screening survey to identify eligible participants, defined as those aged ≥ 18 years who were either expecting a child or had a child aged < 2 years, was distributed to a subset of these panelists based on a computer algorithm designed to optimize the final sample size to 10,000 participants. The exact number of panelists who received the screening survey was not disclosed. Subsequently, we sent email invitations and questionnaires to 3436 male and 14,086 female eligible participants. Of those invited to participate, 10,000 (1953 men and 8047 women) responded between 28 July and 30 August 2021 (response rate: 58.4% for men; 57.1% for women). Of the 1953 male and 8047 female respondents, we excluded 228 men and 720 women with invalid responses, defined as respondents who did not select one of the five possible options in response to a dummy question; those who reported abusing all seven substances on the questionnaire (alcohol, sleeping medications, opioids, sniffing paint thinner, legal high drugs, marijuana, and cocaine/heroin); or those who selected all past medical history listed. These criteria aim to exclude respondents who reported abusing all seven substances listed in the questionnaire or who selected all past medical history options. This exclusion is intended to enhance the reliability and consistency of the data by minimizing the influence of extreme cases on the analysis results. These criteria are standardized and consistently used across the JACSIS study. We also excluded 6 men and 60 women living in poverty and 8 unemployed men (there were no unemployed women) as these factors were considered confounders with a small sample size. We defined poverty as an annual equivalized income of < 1.24 million JPY (8857 USD at 140 JPY/USD), the poverty line in 2018 as defined by the Organisation for Economic Co-operation and Development [28]. In total, we analyzed data from 1711 men (475 expectant fathers and 1236 early-stage fathers) and 7265 women (1630 expectant mothers and 5635 early-stage mothers). Characteristics of these participants are shown in Table 1 and Supplementary Table 1 [see Additional file 1].

**Table 1:** Mean values and proportions for participant characteristics (*n* = 8976)

	Men				Women			
	During pregnancy		Within 2 years after delivery		During pregnancy		Within 2 years after delivery	
	*n* = 475		*n* = 1236		*n* = 1630		*n* = 5635	
	**Mean**	**SD**	**Mean**	**SD**	**Mean**	**SD**	**Mean**	**SD**
Age, years	35.2	5.1	35.5	5.2	31.5	4.5	32.2	4.4
	**n**	**%**	**n**	**%**	**n**	**%**	**n**	**%**
Junior high or high school graduate	56	11.8	134	10.8	249	15.3	885	15.7
Current smoker	97	20.4	266	21.5	34	2.1	244	4.3
Current drinker	261	54.9	737	59.6	63	3.9	1310	23.2
Lives with ≥ 2 children	77	16.2	628	50.8	208	12.8	2576	45.7
Recurrent pregnancy loss	26	5.5	55	4.4	259	15.9	399	7.1
Fetus/infant/child with health problems	19	4.0	40	3.2	27	1.7	198	3.5
History of depression	32	6.7	68	5.5	114	7.0	434	7.7
Paternal leave	-	-	415	33.6	-	-	499	8.9
Maternal leave	-	-	461	37.3	-	-	2420	42.9
Low birth weight infant	-	-	92	7.4	-	-	507	9.0
	**Mean**	**SD**	**Mean**	**SD**	**Mean**	**SD**	**Mean**	**SD**
Recent K6 score	4.3	4.8	3.9	4.8	4.7	5.5	4.3	5.3
	**n**	**%**	**n**	**%**	**n**	**%**	**n**	**%**
Presence of chronic pain	40	8.4	138	11.2	165	10.1	1043	18.5
Presence of death fantasies	54	11.4	98	7.9	142	8.7	598	10.6
Conception method								
NCIP	342	72.0	906	73.3	1017	62.4	3668	65.1
NCUP	37	7.8	126	10.2	221	13.6	1028	34.2
Fertility treatment (SI/OI)	28	5.9	84	6.8	141	8.7	393	7.0
Fertility treatment (IUI)	27	5.7	41	3.3	50	3.1	166	2.9
Fertility treatment (IVF/ICSI)	41	8.6	79	6.4	201	12.3	380	6.7
Dominant site for chronic pain, *n* = 1386	40	(100)	138	(100)	165	(100)	1043	(100)
Head or orofacial	5	(12.5)	11	(8.0)	23	(13.9)	164	(15.7)
Neck or shoulder	17	(42.5)	45	(32.6)	9	(5.5)	197	(18.9)
Chest or abdominal	0	(0)	2	(1.4)	11	(6.7)	87	(8.3)
Back	3	(7.5)	2	(1.4)	6	(3.6)	48	(4.6)
Low back	10	(25.0)	50	(36.2)	66	(40.0)	290	(27.8)
Upper limb, hand, or finger	2	(5.0)	9	(6.5)	2	(1.2)	78	(7.5)
Pelvis	0	(0)	4	(2.9)	26	(15.8)	76	(7.3)
Genitals, urethra, or anus	0	(0)	3	(2.2)	7	(4.2)	21	(2.0)
Hip	0	(0)	0	(0)	10	(6.1)	21	(2.0)
Lower limb	3	(7.5)	12	(8.7)	5	(3.0)	61	(5.8)

ICSI, Intracytoplasmic sperm injection; IUI, Intrauterine insemination; IVF, In vitro fertilization; K6, Kessler psychological distress Scale; NCIP, Naturally conceived intended pregnancy; NCUP, Naturally conceived unintended pregnancy; OI, Ovulation inducer; SD, Standard deviation; SI, Scheduled intercourse

Notably, these expectant mothers may have included women who were considering abortion or even planning to have an abortion at the time they completed their surveys.

### Measures

#### Conceptional methods: exploratory variable

We used a single question to identify conception methods: “Which method was used to achieve your most recent pregnancy?” Respondents chose from six options: (i) naturally conceived intended pregnancy (desired or intended pregnancy planned in terms of timing), (ii) naturally conceived unintended pregnancy (unexpected or undesirable pregnancy), (iii) fertility treatment (scheduled intercourse [SI] or ovulation inducer [OI], combined owing to the relatively low incidence of these among men), (iv) fertility treatment (intrauterine insemination [IUI]), (v) fertility treatment (IVF and intracytoplasmic sperm injection [ICSI]).

#### Psychological distress: outcome measure 1

Psychological distress was defined as a score of ≥ 13 on the Kessler Psychological Distress Scale (K6) [29–31]. Participants were asked “During the past 30 days, about how often did you feel i) nervous, ii) hopeless, iii) restless or fidgety, iv) so depressed that nothing could cheer you up, v) that everything was an effort, and vi) worthless?” Respondents rated the frequency of these feelings as follows: 0 (none of the time), 1 (a little of the time), 2 (some of the time), 3 (most of the time), or 4 (all of the time). Responses are summed to produce a total scale score (range: 0–24). Binarization of the K6 score using the established cutoff point allows for a consistent analytical approach across our various outcome measures, including the binary measures such as death fantasies. This approach facilitates a uniform assessment of recent psychological distress presence or absence, aligning with our other binary outcome variables.

#### Chronic pain: outcome measure 2

Chronic pain was defined as pain that persisted or recurred for longer than 3 months, according to the International Classification of Diseases 11th Revision (ICD-11) [32]. We assessed about the presence of pain symptoms in the past month, with a slight variation in wording between male and female participants. For men, we asked about pain symptoms excluding body aches from a cold (yes or no). For women, we asked about pain symptoms excluding labor pain, pain during childbirth, afterpains, and body aches from a cold (yes or no). Participants who answered ‘Yes’ to this question and indicated that their pain lasted for 3 months or more (≥ 3 and < 6 months, ≥ 6 and < 12 months, or ≥ 1 year) were categorized as having chronic pain. Participants were shown manikins that displayed body regions and asked to identify their dominant pain site.

#### Death fantasies: outcome measure 3

An original single question was used to assess death fantasies: “Have you ever felt that you wanted to die at any time since January 2021?” (yes or no). It should be noted that this single question does not constitute a validated measure of suicide risk, such as the Columbia-Suicide Severity Rating Scale (C-SSRS) [33]. The C-SSRS is a psychometrically validated tool with demonstrated reliability and validity in assessing the full spectrum of suicidal ideation and behaviors, including its ability to differentiate between passive and active ideation, and to predict future suicide attempts [33]. Our single-question measure was designed to assess death fantasies rather than suicidal ideation and should be interpreted with caution, as it does not capture the full intensity or spectrum of suicidal thoughts.

#### Covariates

The following factors potentially related to perinatal parental mental health were considered as covariates (i.e., potential confounding factors). Age, education level, and income can influence stress levels and access to social support, both of which are key in managing perinatal mental health [34, 35]. Smoking and drinking habits, often used as coping mechanisms, may negatively affect perinatal mental health [34]. Pregnancy loss, infant health issues, and a history of depression can increase psychological burden and the risk of mental health problems [34, 36–39]. Furthermore, the availability and duration of parental leave are crucial, with extended leave being associated with improved maternal mental health [40, 41]. Finally, experiences such as having a low birthweight baby or caesarean section may contribute to psychological stress due to unexpected medical challenges [42].

#### For both periods (pregnancy and early parenthood), the following factors were considered

Age (18–24, 25–29, 30–34, 35–39, 40–44, or 45–49 years), educational attainment (junior high or high school graduate, or > high school graduate), smoking status (never-smoker, former smoker, or smoker), drinking status (never-drinker, former drinker, or drinker), equivalized income (quintile), number of children participant lives with (0, 1, or ≤ 2), recurrent pregnancy loss (yes or no), fetal/infant/child with health problems (yes or no), and history of depression (yes or no). Equivalized household income was calculated by dividing the median value of the multiple-choice response to the annual household income question by the square root of the number of people living in the household. Recurrent pregnancy loss was calculated as two or more pregnancy losses. In our questionnaire, the number of pregnancy losses was derived from the difference between the total number of pregnancies and the number of live births. This approach, while commonly used in Japanese epidemiological studies due to the sensitive nature of direct questions regarding miscarriages and induced abortions, may not distinguish between consecutive and non-consecutive losses. Equivalized household income was calculated by dividing the median value of the multiple-choice response to the annual household income question by the square root of the number of people living in the household.

#### For early parenthood, the following factors were considered

Paternal leave (yes or no), maternal leave (yes or no), and low birth weight infant, defined as a body weight of < 2500 g (yes or no). Experience of a cesarean section (yes or no) was used as a covariate for female participants in early parenthood.

Covariate multicollinearity was examined using the variance inflation factor (VIF). The VIF for all covariates was < 3. A VIF of < 5 is generally accepted as indicating a lack of problematic multicollinearity [43].

### Statistical analysis

We used a modified Poisson regression model to compare the relative risk (RR) with 95% confidence intervals (CIs) of recent psychological distress, prevalence of chronic pain, and death fantasies during pregnancy and early parenthood according to conception method compared with naturally conceived intended pregnancy. We used Statistical Analysis Software (SAS; SAS Institute Inc., Cary, NC, USA) and the SAS PROC GENMOD REPEATED statement to estimate the sandwich error using the modified Poisson regression model. Sandwich error is a robust method for estimating standard errors in regression analysis. It is particularly useful when the data are highly variable. It adjusts for irregularities and dependencies in the data, providing more reliable standard error estimates and increasing the accuracy of statistical inference [44].

Model 1 was adjusted for age. Model 2 was adjusted for age, junior high or high school graduate, smoking, drinking, equivalized income, living with ≥ 2 children, recurrent pregnancy loss, infant with health problems, and history of depression. To Model 3 (only for participants in early parenthood) we added paternal leave, maternal leave, and low birth weight infant. Model 4 included the variables from Model 3 plus cesarean section experience (only for chronic pain prevalence among women in early parenthood). The reason cesarean section experience was specifically considered only in Model 4 is that it may have a specific impact on the development of chronic pain. Cesarean section was introduced at a different stage than the other covariates because of its potential impact on the physical recovery process, especially as it may be associated with chronic pain in women. P values < 0.05 (two-tailed tests) were considered statistically significant. All statistical analyses were performed using SAS, Version 9.4.

## Results

### Participant characteristics

Table 1 and Supplemental Table 1 in the Additional file 1 show the participant characteristics. Men whose partners were pregnant were 22–49 years old, men within two years after delivery were 21–49 years old, women during pregnancy were 19–48 years old, and those within two years after delivery were 18–48 years old. The prevalence of recent psychological distress was 10.5% in men expecting a child and 8.7% in men within two years after delivery, whereas it was 7.0% in women during pregnancy and 6.8% in women within two years after delivery. The prevalence of chronic pain was 8.4% in men expecting a child and 11.2% in men within two years after delivery, whereas it was 10.1% in women during pregnancy and 18.5% in women within two years after delivery. The proportion of men experiencing death fantasies was 11.4% among those expecting a child, and 7.9% within two years after delivery. For women, these figures were 8.7% and 10.6%, respectively.

### Recent psychological distress

**Fig. 2:**
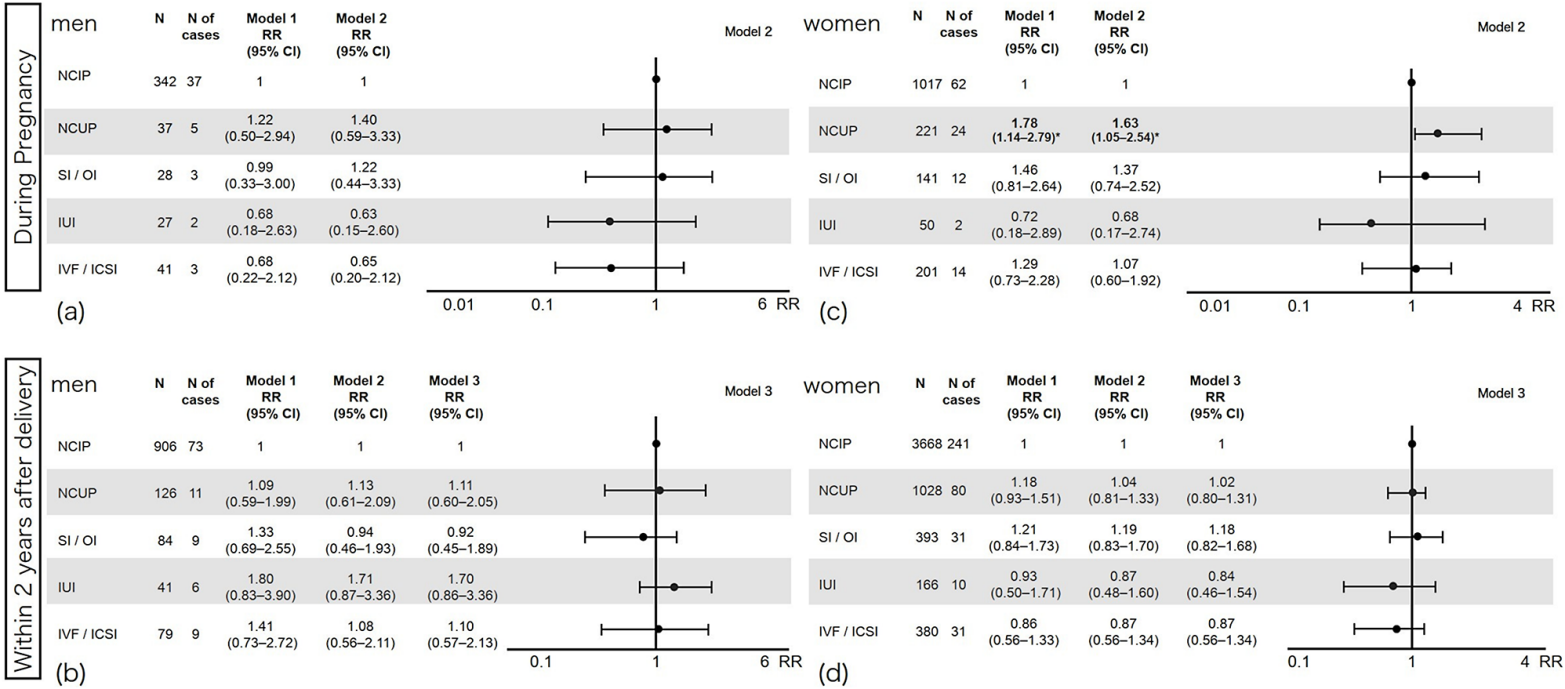
Conception methods and psychological distress. The figure illustrates the relative risks and 95% confidence intervals of psychological distress by conception methods. The X-axis shows the relative risk of psychological distress. The upper left quadrant (**a**) shows male parents whose partners were pregnant; the lower left (**b**) shows male parents in early parenthood; the upper right (**d**) shows female parents during pregnancy; and the lower right (**d**) shows female parents in early parenthood. CI, Confidence interval; ICSI, Intracytoplasmic sperm injection; IUI, Intrauterine insemination; IVF, In vitro fertilization; N, Number; NCIP, naturally conceived intended pregnancy; NCUP, Naturally conceived unintended pregnancy; OI, Ovulation inducer; RR, Relative risk; SI, Scheduled intercourse

Figure 2 shows the association between conception method and recent psychological distress. When compared with naturally conceived intended pregnancy, the following patterns were observed: In male partners during pregnancy (Fig. 2 [a] upper left), there was a non-significant trend toward a higher risk of recent psychological distress in cases of naturally conceived unintended pregnancy and SI/OI (with wide CIs), whereas lower risks were associated with IUI and IVF/ICSI. For men within two years after delivery (Fig. 2 [b] lower left), naturally conceived unintended pregnancy, IUI, and IVF/ICSI were associated with a non-significant trend toward higher risk of recent psychological distress, whereas the risk was lower for SI/OI. However, none of these observed trends reached statistical significance.

Figure 2 (c) shows the results for women during pregnancy. Naturally conceived unintended pregnancy was associated with a significantly increased the risk of recent psychological distress within 30 days (Model 2: RR 1.63, 95% CI 1.05–2.54, *p* < 0.05) among women who were still pregnant. However, this higher risk of recent psychological distress was not observed among women who had already given birth to a naturally conceived unintended pregnancy (Fig. 2 [d] lower right). During pregnancy, there was a non-significant trend towards a higher risk associated with SI/OI, and IUI was linked with a lower risk of recent psychological distress (Fig. 2 [c] upper right). Conversely, within two years after delivery (Fig. 2 [d] lower right), SI/OI exhibited a non-significant trend toward a higher risk of recent psychological distress (with wide CIs), whereas IUI and IVF/ICSI were associated with a trend toward a lower risk of recent psychological distress. However, none of these trends reached statistical significance.

### Chronic pain

**Fig. 3:**
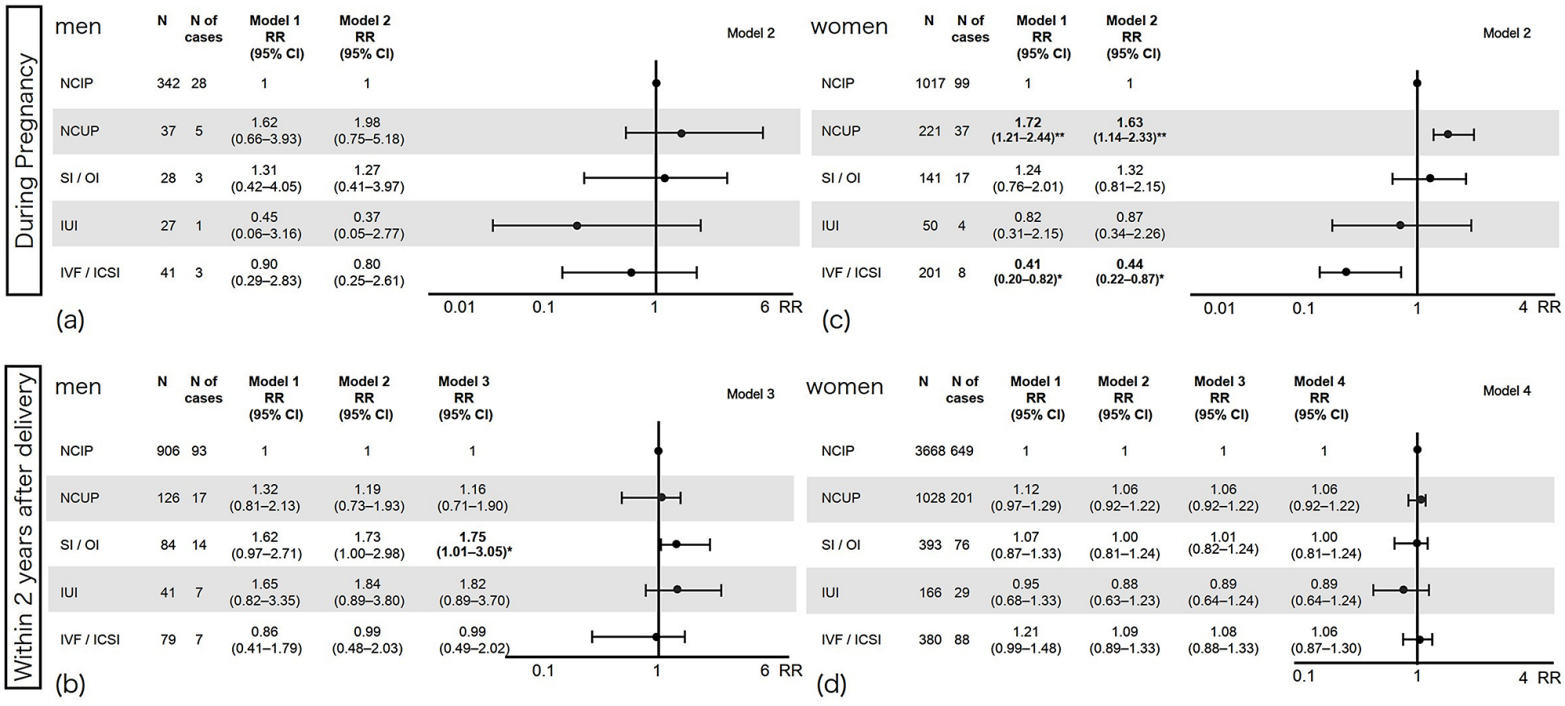
Conception methods and chronic pain. The figure illustrates the relative risks and 95% confidence intervals of prevalence of chronic pain by conception methods. The X-axis shows the relative risk of the prevalence of chronic pain. The upper left quadrant (**a**) shows male parents whose partners were pregnant; the lower left (**b**) shows male parents in early parenthood; the upper right (**c**) shows female parents during pregnancy; and the lower right (**d**) shows female parents in early parenthood. CI, Confidence interval; ICSI, Intracytoplasmic sperm injection; IUI, Intrauterine insemination; IVF, In vitro fertilization; N, Number; NCIP, naturally conceived intended pregnancy; NCUP, Naturally conceived unintended pregnancy; OI, Ovulation inducer; RR, Relative risk; SI, Scheduled intercourse

Figure 3 shows the association between conception method and prevalence of chronic pain. Compared with naturally conceived intended pregnancy, the following patterns were observed.

For male partners during pregnancy (Fig. 3 [a] upper left), the risk of prevalence of chronic pain in cases of naturally conceived unintended pregnancy tended to be twice as high (with wide CIs), and the risk was also higher for SI/OI. However, the risks for chronic pain prevalence with IUI and IVF/ICSI showed a non-significant trend towards being lower (with wide CIs). These results did not reach statistical significance. Conversely, for males during early parenthood (Fig. 3 [b] lower left), the risk of chronic pain prevalence with IVF/ICSI was similar to that of naturally conceived intended pregnancy, whereas naturally conceived unintended pregnancy and IUI were associated with a higher risk. For SI/OI, the risk of chronic pain prevalence was significantly higher (Model 3: RR 1.75, 95% CI 1.01–3.05, *p* < 0.05).

For women during pregnancy (Fig. 3 [c] upper right), the risk of chronic pain prevalence for naturally conceived unintended pregnancy was significantly higher (Model 2: RR 1.63, 95% CI 1.14–2.33, *p* < 0.01). Although not statistically significant, there was a slight, non-significant trend indicating a higher risk of chronic pain prevalence with SI/OI, and a lower risk with IUI. The risk of chronic pain prevalence with IVF/ICSI was significantly lower (Model 2: RR 0.44, 95% CI 0.22–0.87, *p* < 0.05). After delivery (Fig. 3 [d] lower right), the higher risk of chronic pain prevalence observed during pregnancy for naturally conceived unintended pregnancies decreased, indicating that the association was limited to the pregnancy period. While there was a slight tendency for a higher risk of chronic pain prevalence with IVF/ICSI (with wide CIs) and a lower risk with IUI. none of these results reached statistical significance.

### Death fantasies

**Fig. 4:**
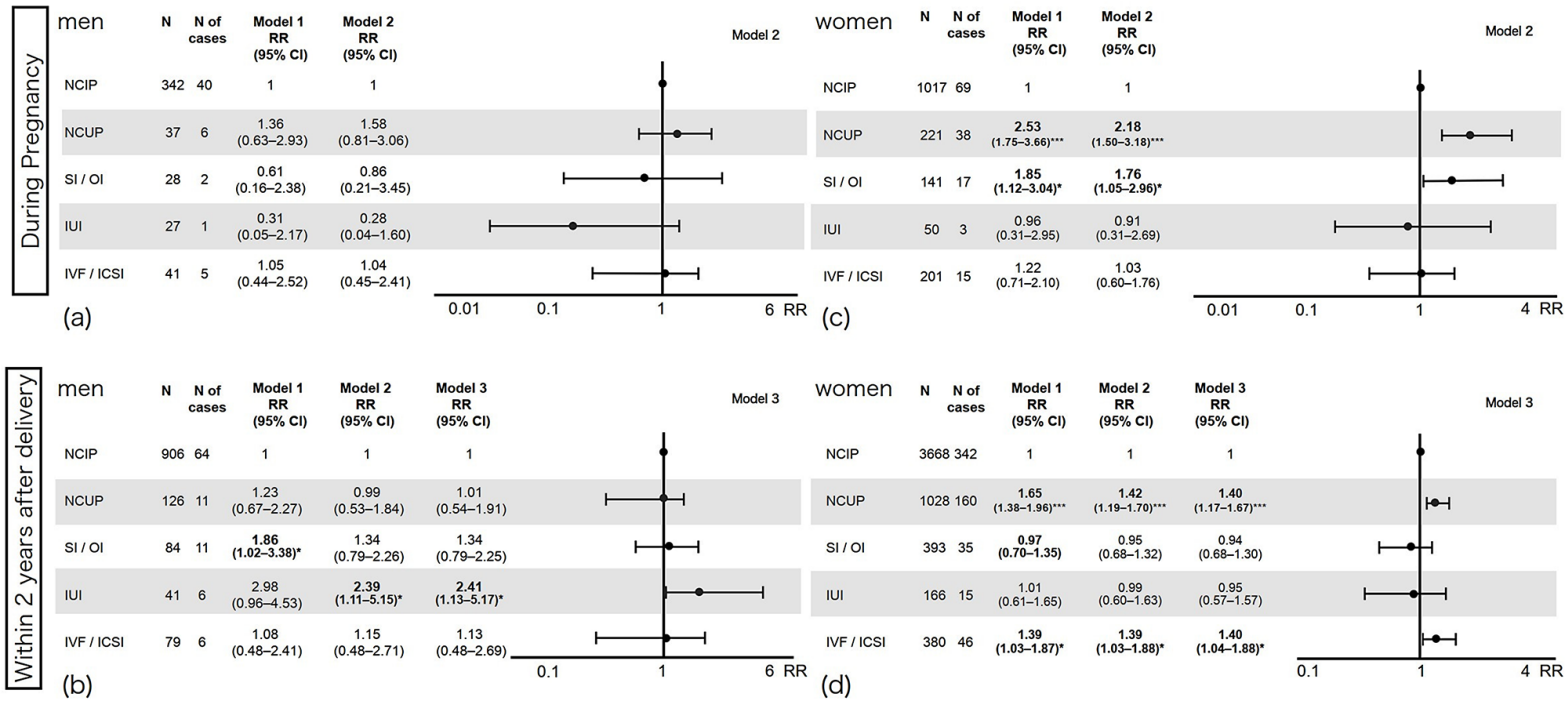
Conception methods and death fantasies. The figure illustrates the relative risks and 95% confidence intervals of death fantasies by conception methods. The X-axis shows the relative risk of death fantasies. The upper left quadrant (**a**) shows male parents whose partners were pregnant; the lower left (**b**) shows male parents in early parenthood; the upper right (**c**) shows female parents during pregnancy; and the lower right (**d**) shows female parents in early parenthood. CI, Confidence interval; ICSI, Intracytoplasmic sperm injection; IUI, Intrauterine insemination; IVF, In vitro fertilization; N, Number; NCIP, Naturally conceived intended pregnancy; NCUP, Naturally conceived unintended pregnancy; OI, Ovulation inducer; RR, Relative risk; SI, Scheduled intercourse

Figure 4 shows the association between conception method and death fantasies. When compared with naturally conceived intended pregnancies, the following patterns were observed.

For men whose partners were pregnant (Fig. 4 [a] upper left), the risk of death fantasies for naturally conceived unintended pregnancy and IVF/ICSI showed a non-significant trend towards being higher (with wide CIs), whereas the risk for SI/OI and IUI showed a non-significant trend towards being lower (with wide CIs). None of these findings were statistically significant. In contrast, men within two years after delivery (Fig. 4 [b] lower left) tended to have a higher risk of death fantasies in cases of naturally conceived unintended pregnancy and IVF/ICSI. The risk of death fantasies also tended to be higher for SI/OI. Men who had undergone IUI had a significantly higher risk of death fantasies (Model 3: RR 2.41, 95% CI 1.13–5.17, *p* < 0.05).

For women during pregnancy (Fig. 4 [c] upper right), the risk of death fantasies was significantly higher for naturally conceived unintended pregnancy (Model 2: RR 2.18, 95% CI 1.50–3.18, *p* < 0.001) and for SI/OI (Model 2: RR 1.76, 95% CI 1.05–2.96, *p* < 0.05). Although not statistically significant, the risk of death fantasies in cases of IUI tended to be lower, whereas the risk for IVF tended to be higher. Conversely, women within two years after delivery (Fig. 4 [d] lower right) had a significantly higher risk of death fantasies for naturally conceived unintended pregnancy (Model 3: RR 1.40, 95% CI 1.17–1.67, *p* < 0.001). The risk of death fantasies for IVF/ICSI was also significantly higher (Model 3: RR 1.40, 95% CI 1.04–1.88, *p* < 0.01). The risk of death fantasies for SI/OI and IUI tended to be roughly equivalent or slightly lower compared with naturally conceived intended pregnancy. None of these findings were statistically significant.

### Covariates

The covariate risks associated with mental health outcomes are shown in Supplemental Tables 2A, 2B, 3A, 3B, 4A and B in Additional File 2. Among the key covariates, history of depression and health problem of fetus/infant/child were strongly associated with mental health risks during pregnancy and up to two years postpartum. Increasing age was linked to higher levels of chronic pain and lower levels of death fantasies in postpartum women. Regarding socioeconomic status, low education was associated with recent psychological distress in both pregnant women and men. Low income showed significant associations with recent psychological distress, chronic pain, and death fantasies in postpartum women. In terms of substance use, smoking was linked to recent psychological distress in postpartum women, as well as to chronic pain and increased death fantasies in men. Alcohol consumption was associated with lower recent psychological distress and death fantasies in men, but in postpartum women, it was linked to increased chronic pain and death fantasies during pregnancy. Notably, several covariates showed stronger associations with mental health outcomes than conception method variables, including unintended pregnancy itself.

## Discussion

We used three markers to assess mental health risks: recent levels of psychological distress, chronic pain as a somatic symptom, and death fantasies. The results of this study suggest that mental health risks associated with unintended natural pregnancies or fertility treatments may vary depending on the time before and after childbirth, as well as the parent’s gender. These findings are consistent with previous meta-analyses showing an increased risk of depression and stress, although variations were observed according to gender and conception method [45, 46]. Notably, women with naturally conceived unintended pregnancies showed a higher tendency for psychological distress during pregnancy, but this distress tended to decrease after childbirth. Moreover, several covariates, such as history of depression, fetal/infant/child health problems, and socioeconomic status, showed stronger associations with mental health outcomes than the conception method variable itself. However, caution is needed in interpreting these results, considering the study’s limitations. These findings highlight the need for flexible and comprehensive mental health support for parents who experience unintended pregnancies or undergo fertility treatments, both during pregnancy and after childbirth. The limitations of this study, including those discussed below, must be taken into account when interpreting these results.

Naturally conceived unintended pregnancy significantly was associated with the risk of recent psychological distress, chronic pain, and death fantasies in women. Notably, recent psychological distress within the past 30 days was higher during pregnancy for women with naturally conceived unintended pregnancies, but lower after delivery, suggesting that the distress may be related to the pregnancy period rather than being prolonged. In men, non-significant trends toward increased risks were observed, but these did not reach statistical significance. Given the confidence intervals, these trends may be clinically important. This finding is consistent with previous studies showing that unintended pregnancy increases the risk of maternal depression and stress, although these risks may diminish as parents adjust after childbirth [47]. These findings support meta-analyses showing an association between naturally conceived unintended pregnancy and increased risk of depression, although this risk may decrease over time [48]. Although this study did not examine whether or not the women were planning to have an abortion during their pregnancy, it is possible that the strong psychological stress of considering whether or not to have an abortion or of having an abortion planned for the near future may have affected the women with naturally conceived unintended pregnancies. They may be associated with feelings of pressure from the male partner, family, or others to have an abortion that is contrary to the woman’s own values and preferences. The mental health risks related to pregnancy loss including abortion on mental health are well known [35–37, 39, 49]. Therefore, it is important to consider the negative impact that unintended pregnancies can have on mental health, especially during pregnancy.

Our findings from this study support recent critiques of the unintended pregnancy framework, as highlighted by Auerbach et al. (2023) [50], suggesting that underlying factors may play a greater role in mental health outcomes than pregnancy intention itself. The strong association between mental health risks during pregnancy and postpartum supports the argument that pre-existing mental health conditions are more predictive of postpartum mental health than pregnancy intention [50]. In addition, our findings add to the evidence that the relationship between unintended pregnancy and mental health is complex and multifaceted [50]. Pre-existing mental health problems may influence both the perception of a pregnancy as unintended and the subsequent postpartum challenges [51]. This perspective shifts the focus from the unintended pregnancy itself to the broader context of pre-pregnancy mental health conditions. This interpretation is consistent with the findings of comprehensive reviews, as highlighted in a previous report, which concluded that abortion is not a direct cause of mental health problems [49]. Instead, associations often reflect pre-existing or concurrent mental health conditions associated with unintended pregnancy or abortion. These findings underscore the importance of addressing the broader psychosocial factors that shape both pregnancy intentions and mental health outcomes. In light of these findings, future research should take a more nuanced approach that includes the broader social and structural context, including pre-existing mental health conditions, socioeconomic factors, and other potential confounders. This comprehensive approach will better capture the interplay between pregnancy intentions and mental health outcomes.

We found that during pregnancy, SI/OI use was generally associated with higher mental health risks for both men and women, compared with individuals who had children through naturally conceived intended pregnancies. Notably, men who used SI/OI during early parenthood also demonstrated a significantly higher risk of chronic pain and death fantasies than men who experienced a naturally conceived intended pregnancy. Conversely, women who used SI/OI during early parenthood did not show an increased mental health risk. The reasons for the observed differences in mental health risks, with men showing an increased risk of chronic pain and death fantasies while women did not, are unclear, but the stress associated with timed intercourse may place additional strain on relationships. Women may be less affected during early parenthood, when the pressure to time intercourse has subsided.

This study is the first to categorize assisted reproductive technology (ART) into IUI and IVF/ICSI to examine their differential effects on mental health risks in male and female parents in early parenthood. During pregnancy, participants who had children using ART demonstrated lower or the same mental health risks as those who had children through naturally conceived intended pregnancy. It is reasonable to assume that individuals who have had a child after enduring the difficulties of infertility and the physical and financial burdens of ART have lower mental health risk than those who conceived easily without such challenges. Men who used IUI during early parenthood showed an unexpected increase in risk of death fantasies, whereas IVF had no effect on mental health outcomes at any stage. For women, ART did not generally increase mental health risks, with the exception of postpartum death fantasies among IVF/ICSI users. The reasons why IUI had a greater effect on men during early parenthood remain unclear.

Differences in stress experienced by men undergoing IUI and IVF/ICSI may reflect factors such as the timing of semen sample collection, which member of the couple is infertile, and differences in men’s perception of their role in fertility treatment. IUI semen sample collection is typically timed to coincide with ovulation, which is predicted by monitoring ovarian follicle growth. In contrast, IVF tends to be more predictable, with semen collection typically timed to a controlled cycle. Additionally, when IUI is used, there is often no cause of female infertility (e.g., tubal occlusion), which may increase pressure on the male partner. Furthermore, in Japan, fertility treatments typically progress from SI/OI to IUI (up to six attempts) and finally to IVF [52], which may increase psychological relief for men after successful IVF attempts. A previous study also showed that women who used IVF or ICSI experienced less recent psychological distress than those who used OI or male artificial insemination [53], which is consistent with our findings.

This study’s approach of investigating both naturally conceived unintended pregnancies and those resulting from fertility treatments offers a more comprehensive understanding of how pregnancy intention affects mental health. While these scenarios represent opposite ends of the pregnancy intentionality spectrum, they share certain mental health impacts. Women with unintended pregnancies experienced higher risks of psychological distress, chronic pain, and death fantasies during pregnancy, likely due to unexpected stressors and lack of preparation associated with unplanned pregnancies. In contrast, women who underwent IVF/ICSI showed a significantly lower risk of chronic pain during pregnancy, possibly reflecting the extensive medical support and psychological preparation involved in fertility treatments.

However, despite these differences, both groups also share some common mental health challenges. For example, while the IVF/ICSI group exhibited a lower risk of chronic pain during pregnancy, they showed a higher risk of suicidal ideation and death fantasies during early parenthood. This might reflect a mismatch between the expectations formed during long-term fertility treatment and the realities of parenting, or a lack of post-treatment support. Similarly, unintended pregnancies, while associated with heightened stress during pregnancy, may lead to ongoing relationship tensions and adjustment difficulties in the postpartum period.

Both unintended pregnancy and fertility treatment can contribute to mental health risks through direct stressors, such as the emotional and financial burdens of treatment or the strain of an unplanned pregnancy. Relationship deterioration of fertility treatment is a common reason for discontinuation [6]. These stressors may also lead to indirect impacts, such as relationship strain and disruptions to work or social life. While the immediate stress from fertility treatments after childbirth, relationship tensions and difficulties in accepting an unintended pregnancy could persist, potentially contributing to mental health risks in the postpartum period. Further research is needed to explore these shared and distinct pathways.

This is the first study to indicate that men who use IVF are not associated with increased psychological risk during pregnancy and early parenthood. These findings provide psychological reassurance to both individuals and health care providers. However, men undergoing SI/OI and IUI may face increased mental health risks during early parenthood. Targeted mental health support is critical for male parents using these treatments, as well as for female parents coping with unintended pregnancy.

There are several limitations that should be considered when interpreting these findings. First, the data do not necessarily represent all residents in Japan, and respondents to web-based surveys may differ in important ways from the general population. However, the data were obtained by sending random invitations to many registrants across Japan, and therefore have some validity regarding representation. Second, this was a cross-sectional study, so temporal causality cannot be assumed. For instance, individuals at psychological risk may be more likely to experience infertility and to use ART [54]. However, this seems unlikely because mental health risk among participants who used IVF did not increase during pregnancy and early parenthood. Moreover, the results remained unchanged after the model was adjusted for depression history. Third, this study was conducted during the COVID-19 pandemic in 2021. The pandemic may have exacerbated negative emotions and thus increased the risk of mental health problems. Fourth, we did not consider the duration of fertility treatment. Generally, even with the same conception method, patient burden increases with longer duration, so length of treatment may have affected the findings. Fifth, while we accounted for pregnancy loss by calculating the difference between the number of pregnancies and live births, this approach does not allow us to distinguish between induced abortions, miscarriages, and stillbirths. In addition, our definition of recurrent pregnancy loss does not take into account whether losses were consecutive, which is the standard criterion. This limitation may affect the precision of our findings regarding the impact of pregnancy loss on psychological outcomes. Sixth, the potential overlap of two different time periods—during pregnancy and postpartum—for women who gave birth or had an abortion in 2021, which may affect the interpretation of the results. Additionally, we did not collect data on whether pregnant women were considering abortion, planning to abort, or facing pressure to abort from others, which may have contributed to psychological distress across all three outcome measures. Furthermore, the measure used to assess death fantasies was not a validated scale for suicidal risk, which may limit the interpretation of the results in this area. Seventh, the study used a binary measure of pregnancy intention rather than a specific scale, which may have missed the more nuanced aspects of participants’ attitudes. Respondents were asked directly whether the pregnancy was considered “intended” or “unintended.” This binary approach may not fully capture the complexity of pregnancy intentions. As Santelli et al. (2009) suggest [55], pregnancy intentions are multidimensional and include aspects such as the timing of pregnancy, emotional reactions, and partner-specific factors. The lack of a more detailed measure in our study limits the depth of insight into participants’ pregnancy intentions and may obscure important variations that could affect the observed outcomes.

## Conclusions

Markers for mental health risks associated with unintended naturally conceived pregnancy or fertility treatment varied before and after live births, as well as according to the parent’s gender. Notably, levels of recent psychological distress within the past 30 days were higher during pregnancy for women with naturally conceived unintended pregnancies, but declined after delivery. Flexible and comprehensive mental health support is needed both during pregnancy and within two years after delivery for individuals who conceived unintentionally or through fertility treatment.

## Electronic supplementary material

Below is the link to the electronic supplementary material.

Supplementary Material 1

Supplementary Material 2

## Data Availability

The data used in this study are not publicly available because they contain personally identifiable or potentially sensitive patient information. Determining whether data are truly non-identifying can be challenging, especially when combining multiple low-frequency responses, which could lead to participant identification. For this reason, we obtained approval from the Research Ethics Committee of the Osaka International Cancer Institute under the condition that data would not be redistributed without explicit participant consent. However, we are open to sharing de-identified data, pending consultation with the Ethics Committee to ensure compliance with ethical guidelines. Researchers interested in accessing the data should contact Dr. Takahiro Tabuchi at tabuchitak@gmail.com. Requests will be reviewed on a case-by-case basis.

## Abbreviations

IVF: In vitro fertilization
COVID: Coronavirus Disease
JACSIS: The 2021 Japan Coronavirus Disease-19 and Society Internet Survey
SI: Scheduled intercourse
OI: Ovulation inducer
IUI: Intrauterine insemination
ICSI: Intracytoplasmic sperm injection
K6: Kessler Psychological Distress Scale
ICD-11: International Classification of Diseases 11th Revision
VIF: Variance inflation factor
RR: Relative risk
CI: Confidence interval
SAS: Statistical Analysis Software
ART: Assisted reproductive technology
