# Supplementary material for: Mental health risks in pregnancy and early parenthood among male and female parents following unintended pregnancy or fertility treatment: a cross-sectional observational study
Source: BMC Pregnancy Childbirth. 2024 Dec 26;24:860. doi: 10.1186/s12884-024-07082-x (PMC11670436; doi:10.1186/s12884-024-07082-x)
Supplement: Supplementary file 1 — Supplementary Material 1 [file 12884_2024_7082_MOESM1_ESM.pdf]

Supplementary Table 1. Participant characteristics (n = 8976)

	Men, n = 1711				Women, n= 7265			
	During pregnancy (n=475)		Within 2 years after delivery (n=1236)		During pregnancy (n=1630)		Within 2 years after delivery (n=5635)	
	n	%	n	%	n	%	n	%
**Age, years**								
18–19	-	-	-	-	1	0.1	3	0.1
20–24	5	1.1	8	0.6	71	4.4	176	3.1
25–29	51	10.7	146	11.8	511	31.3	1465	26.0
30–34	171	36.0	411	33.3	629	38.6	2294	40.7
35–39	157	33.1	415	33.6	343	21.0	1430	25.4
40–44	67	14.1	177	14.3	72	4.4	250	4.4
45–49	24	5.1	79	6.4	3	0.2	17	0.3
**Educational attainment**								
Less than high school	1	0.2	0	0.0	11	0.7	30	0.5
High school	55	11.6	134	10.8	238	14.6	855	15.2
Vocational school	46	9.7	114	9.2	289	17.7	1093	19.4
Junior or technical college	10	2.1	30	2.4	176	10.8	685	12.2
University	285	60.0	780	63.1	860	52.8	2781	49.4
Graduate school	74	15.6	176	14.2	51	3.1	174	3.1
Others	4	0.8	2	0.2	5	0.3	17	0.3
**Marital status**								
Single	0	0	0	0	21	1.3	38	0.7
Married or common law marriage (living together)	471	99.2	1232	99.7	1558	95.6	5431	96.4
Married or common law marriage (separated by reasons business reason)	0	0	0	0	22	1.3	101	1.8
Married or common law (separated by reasons other than business reasons)	4	0.8	4	0.3	20	1.2	34	0.6
Divorced/widowed	0	0	0	0	9	0.6	31	0.6

**Smoking status**								
Never smoker	211	44.4	581	47.0	1213	74.4	4080	72.4
Former smoker	167	35.2	389	31.5	383	23.5	1311	23.3
Smoker	97	20.4	266	21.5	34	2.1	244	4.3
**Alcohol consumption habit**								
Never	65	13.7	145	11.7	364	22.3	938	16.6
Former drinker	149	31.4	354	28.6	1203	73.8	3387	60.1
Drinker	261	54.9	737	59.6	63	3.9	1310	23.2
**Employment status**								
Company executive	25	5.3	60	4.9	31	1.9	135	2.4
Owner of family operated business	14	2.9	43	3.5	26	1.6	78	1.4
Employee of family operated business	2	0.4	4	0.3	7	0.4	37	0.7
Management level employee	82	17.3	193	15.6	101	6.2	339	6.0
Full-time employee	337	70.9	913	73.9	639	39.2	2249	39.9
Contract employee	12	2.5	11	0.9	99	6.1	257	4.6
Part-time employee/on-the-side worker	1	0.2	3	0.2	229	14.0	695	12.3
Student	2	0.4	1	0.1	0	0.0	2	0.0
Full-time homemaker	0	0	8	0.6	160	9.8	714	12.7
**Employment status of partner**								
Company executive	8	1.7	17	1.4	142	8.7	457	8.1
Owner of family operated business	13	2.7	20	1.6	93	5.7	333	5.9
Employee of family operated business	3	0.6	13	1.1	10	0.6	32	0.6
Management level employee	24	5.0	58	4.7	295	18.1	1118	19.8
Full-time employee	235	49.3	518	41.9	1002	61.5	3405	60.4
Contract employee	17	3.6	38	3.1	22	1.3	84	1.5
Part-time employee/on-the-side worker	43	9.0	132	10.7	13	0.8	57	1.0
Student	0	0.0	2	0.2	1	0.1	11	0.2
Full-time homemaker	87	18.2	324	26.2	15	0.9	47	0.8
Unemployed	45	9.4	114	9.2	7	0.4	22	0.4
**Equivalized household income, million Japanese yen**								
Q1: women –3.70; men –4.24	73	15.4	261	21.1	218	13.4	1187	21.1
Q2: women 3.71–5.77; men 4.25–6.52	81	17.1	264	21.4	303	18.6	1181	21.0
Q3: women 5.79–9.40; men 6.57–9.53	89	18.7	256	20.7	357	21.9	1088	19.3
Q4: women 9.41–52.00; men 9.55–16.74	119	25.1	224	18.1	396	24.3	1070	19.0
Q5: women 52.05–; men 16.80–	113	23.8	230	18.6	356	21.8	1109	19.7
Missing	0	0	1	0.1	0	0	0	0

**Number of children living together**								
None	241	50.7	0	0	974	59.8	0	0
1	157	33.1	608	49.2	448	27.5	3059	54.3
≥2	77	16.2	628	50.8	208	12.8	2576	45.7
**Number of pregnancy loss**								
Zero	147	30.9	1046	84.6	351	21.5	4347	77.1
Once	302	63.6	135	10.9	1020	62.6	889	15.8
Twice or more	26	5.5	55	4.4	259	15.9	399	7.1
**Fetus/Infant with health problems**								
None	456	96.0	1196	96.8	1603	98.3	5437	96.5
Yes	19	4.0	40	3.2	27	1.7	198	3.5
**History of depression**								
None	443	93.3	1168	94.5	1516	93.0	5201	92.3
Yes	32	6.7	68	5.5	114	7.0	434	7.7
**Paternal leave**								
No	-	-	813	65.8	-	-	5067	89.9
Unemployed prior to pregnant/childbirth	-	-	8	0.6	-	-	69	1.2
Yes	-	-	415	33.6	-	-	499	8.9
**Maternal leave**								
No	-	-	476	38.5	-	-	2086	37.0
Unemployed prior to pregnant/childbirth	-	-	299	24.2	-	-	1129	20.0
Yes	-	-	461	37.3	-	-	2420	42.9
**Gestational week**								
Premature birth	-	-	160	12.9	-	-	336	6.0
Full term birth	-	-	978	79.1	-	-	5211	92.5
Postmature birth	-	-	98	7.9	-	-	88	1.6
**Birth weight**								
Low birth weight infant	-	-	92	7.4	-	-	507	9.0
Normal birth weight infant	-	-	1134	91.7	-	-	5088	90.3
High birth weight infant	-	-	10	0.8	-	-	40	0.7
	**Means**	**SD**	**Means**	**SD**	**Means**	**SD**	**Means**	**SD**
**Recent K6 score (3-15 points)**	4.7	5.5	4.3	5.3	4.3	4.8	3.9	4.8
	**n**	**%**	**n**	**%**	**n**	**%**	**n**	**%**
**Presence of chronic pain**								
None	435	91.6	1098	88.8	1465	89.9	4592	81.5
Yes	40	8.4	138	11.2	165	10.1	1043	18.5

**Presence of death fantasies**								
None	421	88.6	1138	92.1	1488	91.3	5037	89.4
Yes	54	11.4	98	7.9	142	8.7	598	10.6
**Conception method**								
NCIP	342	72.0	906	73.3	1017	62.4	3668	65.1
NCUP	37	7.8	126	10.2	221	13.6	1028	18.2
Fertility treatment (SI)	19	4.0	64	5.2	95	5.8	276	4.9
Fertility treatment (OI)	9	1.9	20	1.6	46	2.8	117	2.1
Fertility treatment (IUI)	27	5.7	41	3.3	50	3.1	166	2.9
Fertility treatment (IVF/ICSI)	41	8.6	79	6.4	201	12.3	380	6.7

K6, Kessler psychological distress scale; ICSI, Intracytoplasmic sperm injection; IUI, Intrauterine insemination; IVF, In vitro fertilization; NCIP, Naturally conceived intended pregnancy; NCUP, Naturally conceived unintended pregnancy; OI, Ovulation inducer; Q, Quintile; SD, Standard deviation; SI, Scheduled intercourse.
