# Supplementary material for: Mental health risks in pregnancy and early parenthood among male and female parents following unintended pregnancy or fertility treatment: a cross-sectional observational study
Source: BMC Pregnancy Childbirth. 2024 Dec 26;24:860. doi: 10.1186/s12884-024-07082-x (PMC11670436; doi:10.1186/s12884-024-07082-x)
Supplement: Supplementary file 2 — Supplementary Material 2 [file 12884_2024_7082_MOESM2_ESM.pdf]

Supplementary Table 2A. The association between the way of getting pregnant and recent psychological distress during pregnancy

	Men, n = 475			Women, n = 1630		
	Recent psychological distress n = 50			Recent psychological distress n = 114		
			Model 2			Model 2
	n	Number of cases	RR (95% CI)	n	Number of cases	RR (95% CI)
Expected spontaneous pregnancy	342	37	1	1017	62	1
Unexpected spontaneous pregnancy	37	5	1.40 (0.59–3.33)	221	24	**1.63 (1.05–2.54)***
Fertility treatment (SI/OI)	28	3	1.22 (0.44–3.33)	141	12	1.37 (0.74–2.52)
Fertility treatment (IUI)	27	2	0.63 (0.15–2.60)	50	2	0.68 (0.17–2.74)
Fertility treatment (IFV/ICSI)	41	3	0.65 (0.20–2.12)	201	14	1.07 (0.60–1.92)
Aging (10 years increment)			0.92 (0.71–1.18)			0.84 (0.70–1.01)
Junior high or high school graduate			1.90 (0.94–3.85)			**1.59 (1.03–2.46)***
Former smoker (vs. Never smoker)			1.03 (0.55–1.92)			1.24 (0.82–1.87)
Current smoker (vs. Never smoker)			1.27 (0.63–2.53)			1.03 (0.38–2.77)
Former drinker (vs. Never drinker)			0.97 (0.45–2.11)			0.93 (0.60–1.45)
Current drinker (vs. Current drinker)			0.72 (0.33–1.57)			1.62 (0.76–3.48)
Equivalized household income Q4 (vs. Q5)			1.03 (0.53–1.99)			1.33 (0.79–2.24)
Equivalized household income Q3 (vs. Q5)			1.04 (0.51–2.12)			1.00 (0.58–1.73)
Equivalized household income Q2 (vs. Q5)			0.48 (0.19–1.25)			0.97 (0.56–1.69)
Equivalized household income Q1 (vs. Q5)			0.55 (0.21–1.42)			0.60 (0.31–1.18)
A children living together			1.51 (0.87–2.64)			0.89 (0.58–1.36)

Two or more children living together	0.96 (0.36–2.52)	0.90 (0.49–1.65)
Recurrent pregnancy loss	1.36 (0.57–3.29)	1.10 (0.68–1.77)
Health problem of fetal/infant/child	**2.58 (1.39–4.82)****	**2.35 (1.14–4.88)***
History of depression	**2.17 (1.11–4.23)***	**3.37 (2.18–5.19)*****

Model 2: Adjusted for age, junior high or high school graduate, smoking, drinking, equivalized income, number of children living together, recurrent pregnancy loss, baby's health problem, and history of depression.

Abbreviations: CI, confidence interval; ICSI, Intracytoplasmic sperm injection; IUI, Intrauterine insemination; IVF, In–vitro fertilization; OI, Ovulation inducer, RR, Relative risk; SI, Scheduled intercourse.

*p <0.05, **p <0.01, ***p <0.001. n = 2105

Supplementary Table 2B. The association between the way of getting pregnant and recent psychological distress within 2 years after delivery

	Men, n = 1236			Women, n = 5635		
	Recent psychological distress n = 108			Recent psychological distress n = 383		
			Model 3			Model 3
	n	Number of cases	RR (95% CI)	n	Number of cases	RR (95% CI)
Expected spontaneous pregnancy	906	73	1	3668	241	1
Unexpected spontaneous pregnancy	126	11	1.11 (0.60–2.05)	1028	80	1.02 (0.80–1.31)
Fertility treatment (SI/OI)	84	9	0.92 (0.45–1.89)	393	31	1.18 (0.82–1.68)
Fertility treatment (IUI)	41	6	1.70 (0.86–3.36)	166	10	0.84 (0.46–1.54)
Fertility treatment (IFV/ICSI)	79	9	1.10 (0.57–2.13)	380	31	0.87 (0.56–1.34)
Aging (10 years increment)			1.04 (0.88–1.24)			0.99 (0.89–1.11)
Junior high or high school graduate			0.96 (0.54–1.7)			0.95 (0.74–1.23)
Former smoker (vs. Never smoker)			1.29 (0.82–2.02)			**1.32 (1.05–1.65)***
Current smoker (vs. Never smoker)			1.20 (0.73–1.97)			**1.76 (1.22–2.55)****
Former drinker (vs. Never drinker)			**0.41 (0.26–0.66)*****			**0.73 (0.57–0.95)***
Current drinker (vs. Current drinker)			**0.35 (0.22–0.56)*****			0.75 (0.56–1.01)
Equivalized household income Q4 (vs. Q5)			1.05 (0.57–1.92)			1.29 (0.91–1.84)
Equivalized household income Q3 (vs. Q5)			1.25 (0.71–2.20)			**1.45 (1.03–2.05)***
Equivalized household income Q2 (vs. Q5)			0.90 (0.49–1.68)			**1.52 (1.09–2.11)***
Equivalized household income Q1 (vs. Q5)			1.00 (0.55–1.83)			**2.00 (1.45–2.76)*****
A child living together			1.11 (0.76–1.64)			**1.27 (1.03–1.56)***

Two or more children living together	–	–
Recurrent pregnancy loss	1.21 (0.59–2.46)	0.84 (0.56–1.24)
Health problem of fetal/infant/child	**3.38 (2.04–5.59)*** **	1.23 (0.79–1.91)
History of depression	**3.55 (2.21–5.71)*** **	**3.19 (2.54–3.99)*** **
Paternal leave	0.85 (0.57–1.26)	1.06 (0.76–1.48)
Maternal leave	0.91 (0.63–1.32)	**0.72 (0.58–0.90)** **
Low birth weight infant	0.82 (0.40–1.69)	1.34 (1.00–1.80)

Model 3: Adjusted for above variables plus paternal leave, maternal leave of partner, and low birth weight infant.

Abbreviations: CI, confidence interval; ICSI, Intracytoplasmic sperm injection; IUI, Intrauterine insemination; IVF, In–vitro fertilization; OI, Ovulation inducer, RR, Relative risk; SI, Scheduled intercourse.

*p <0.05, **p <0.01, ***p<0.001. n = 6871

Supplementary Table 3A. The association between the way of getting pregnant and prevalence of chronic pain during pregnancy

	Men, n = 475			Women, n = 1630		
	Chronic pain, n = 40			Chronic pain, n = 165		
			Model 2			Model 2
	n	Number of cases	RR (95% CI)	n	Number of cases	RR (95% CI)
Expected spontaneous pregnancy	342	28	1	1017	99	1
Unexpected spontaneous pregnancy	37	5	1.98 (0.75–5.18)	221	37	**1.63 (1.14–2.33)****
Fertility treatment (SI/OI)	28	3	1.27 (0.41–3.97)	141	17	1.32 (0.81–2.15)
Fertility treatment (AIH)	27	1	0.37 (0.05–2.77)	50	4	0.87 (0.34–2.26)
Fertility treatment (IVF)	41	3	0.80 (0.25–2.61)	201	8	**0.44 (0.22–0.87)***
Aging (10 years increment)			0.92 (0.69–1.22)			0.93 (0.79–1.10)
Junior high or high school graduate			0.15 (0.02–1.24)			0.90 (0.60–1.34)
Former smoker (vs. Never smoker)			1.19 (0.57–2.48)			1.06 (0.75–1.51)
Current smoker (vs. Never smoker)			**2.18 (1.06–4.49)***			1.20 (0.53–2.71)
Former drinker (vs. Never drinker)			0.84 (0.34–2.10)			1.41 (0.94–2.10)
Current drinker (vs. Current drinker)			0.61 (0.24–1.58)			1.22 (0.54–2.79)
Equivalized household income Q4 (vs. Q5)			1.57 (0.71–3.48)			1.13 (0.72–1.79)
Equivalized household income Q3 (vs. Q5)			1.05 (0.41–2.65)			1.29 (0.83–2.03)
Equivalized household income Q2 (vs. Q5)			0.40 (0.12–1.29)			0.89 (0.54–1.45)
Equivalized household income Q1 (vs. Q5)			0.94 (0.32–2.78)			1.38 (0.86–2.22)
A child living together			1.09 (0.56–2.13)			**1.42 (1.03–1.97)***
Two or more children living together			1.33 (0.58–3.05)			1.33 (0.88–2.03)

Recurrent pregnancy loss	1.82 (0.73–4.53)	1.33 (0.93–1.89)
Health problem of fetal/infant/child	1.93 (0.70–5.28)	1.39 (0.53–3.67)
History of depression	2.14 (0.91–5.03)	1.57 (0.98–2.51)

Model 2: Adjusted for age, junior high or high school graduate, smoking, drinking, equivalized income, number of children living together, recurrent pregnancy loss, baby's health problem, and history of depression.

Abbreviations: CI, confidence interval; ICSI, Intracytoplasmic sperm injection; IUI, Intrauterine insemination; IVF, In–vitro fertilization; OI, Ovulation inducer, RR, Relative risk; SI, Scheduled intercourse.

*p <0.05, **p <0.01. n = 2105

Supplementary Table 3B. The association between the way of getting pregnant and prevalence of chronic pain within 2 years after delivery

	Men, n = 1236			Women, n = 5635		
	Chronic pain, n = 40			Chronic pain, n = 165		
			Model 3			Model 4
	n	Number of cases	RR (95% CI)	n	Number of cases	RR (95% CI)
Expected spontaneous pregnancy	906	93	1	3668	649	1
Unexpected spontaneous pregnancy	126	17	1.16 (0.71–1.90)	1028	201	1.06 (0.92–1.22)
Fertility treatment (SI/OI)	84	14	**1.75 (1.01–3.05)***	393	76	1.00 (0.81–1.24)
Fertility treatment (AIH)	41	7	1.82 (0.89–3.70)	166	29	0.89 (0.64–1.24)
Fertility treatment (IVF)	79	7	0.99 (0.49–2.02)	380	88	1.06 (0.87–1.30)
Aging (10 years increment)			1.04 (0.90–1.2)			**1.12 (1.05–1.19)*****
Junior high or high school graduate			0.98 (0.60–1.63)			0.93 (0.79–1.09)
Former smoker (vs. Never smoker)			0.98 (0.67–1.42)			**1.24 (1.09–1.40)*****
Current smoker (vs. Never smoker)			1.03 (0.68–1.55)			0.95 (0.71–1.28)
Former drinker (vs. Never drinker)			1.62 (0.81–3.23)			**1.66 (1.36–2.01)*****
Current drinker (vs. Current drinker)			**1.98 (1.02–3.85)***			**1.67 (1.35–2.07)*****
Equivalized household income Q4 (vs. Q5)			0.94 (0.55–1.63)			1.08 (0.91–1.29)
Equivalized household income Q3 (vs. Q5)			1.28 (0.77–2.12)			1.03 (0.86–1.23)
Equivalized household income Q2 (vs. Q5)			0.95 (0.56–1.61)			1.18 (0.99–1.41)
Equivalized household income Q1 (vs. Q5)			1.01 (0.61–1.69)			**1.21 (1.01–1.44)***
A child living together			**0.66 (0.46–0.93)***			**1.34 (1.19–1.51)*****
Two or more children living together			–			–

Recurrent pregnancy loss	1.07 (0.53–2.19)	1.13 (0.93–1.37)
Health problem of fetal/infant/child	1.41 (0.63–3.18)	1.23 (0.95–1.57)
History of depression	**1.88 (1.11–3.19)***	**1.65 (1.41–1.93)*****
Paternal leave	0.97 (0.69–1.37)	1.03 (0.86–1.24)
Maternal leave	0.73 (0.52–1.03)	**1.12 (1.01–1.26)***
Low birth weight infant	0.68 (0.32–1.45)	1.04 (0.86–1.25)
Cesarean section	–	1.13 (0.99–1.29)

Model 3: Adjusted for above variables plus paternal leave, maternal leave of partner, and low birth weight infant.

Model 4: Adjusted for age, junior high or high school graduate, smoking, drinking, equivalized income, number of children living together, recurrent pregnancy loss, baby's health problem, history of depression, and variables plus cesarean section.

Abbreviations: CI, confidence interval; ICSI, Intracytoplasmic sperm injection; IUI, Intrauterine insemination; IVF, In–vitro fertilization; OI, Ovulation inducer, RR, Relative risk; SI, Scheduled intercourse.

*p < 0.05, ***p <0.001. n = 6871

Supplementary Table 4A. The association between the way of getting pregnant and death fantasies during pregnancy

	Men, n = 1711			Women, n = 7265		
	Death fantasies n = 54			Death fantasies n = 142		
			Model 2			Model 2
	n	Number of cases	RR (95% CI)	n	Number of cases	RR (95% CI)
Expected spontaneous pregnancy	342	40	1	1017	69	1
Unexpected spontaneous pregnancy	37	6	1.58 (0.81–3.06)	221	38	**2.18 (1.50–3.18)*** **
Fertility treatment (SI/OI)	28	2	0.86 (0.21–3.45)	141	17	**1.76 (1.05–2.96)***
Fertility treatment (AIH)	27	1	0.28 (0.04–1.60)	50	3	0.91 (0.31–2.69)
Fertility treatment (IVF)	41	5	1.04 (0.45–2.41)	201	15	1.03 (0.60–1.76)
Aging (10 years increment)			0.95 (0.77–1.19)			0.87 (0.73–1.04)
Junior high or high school graduate			1.03 (0.50–2.15)			1.26 (0.84–1.90)
Former smoker (vs. Never smoker)			**2.26 (1.22–4.16)****			1.18 (0.81–1.72)
Current smoker (vs. Never smoker)			**2.33 (1.17–4.66)***			1.05 (0.45–2.44)
Former drinker (vs. Never drinker)			1.19 (0.60–2.33)			1.36 (0.88–2.11)
Current drinker (vs. Current drinker)			0.56 (0.27–1.17)			**2.28 (1.12–4.64)***
Equivalized household income Q4 (vs. Q5)			1.22 (0.60–2.5)			0.85 (0.53–1.38)
Equivalized household income Q3 (vs. Q5)			1.22 (0.61–2.43)			0.79 (0.49–1.29)
Equivalized household income Q2 (vs. Q5)			0.90 (0.39–2.03)			1.20 (0.77–1.89)
Equivalized household income Q1 (vs. Q5)			1.66 (0.78–3.54)			1.02 (0.59–1.74)
A child living together			1.11 (0.67–1.84)			0.96 (0.67–1.39)

Two or more children living together	0.54 (0.21–1.40)	0.61 (0.34–1.10)
Recurrent pregnancy loss	1.41 (0.54–3.69)	1.21 (0.80–1.81)
Health problem of fetal/infant/child	**2.10 (1.17–3.78)***	**2.61 (1.38–4.92)****
History of depression	**3.67 (2.21–6.10)*****	**3.43 (2.39–4.94)*****

Model 2: Adjusted for age, junior high or high school graduate, smoking, drinking, equivalized income, number of children living together, recurrent pregnancy loss, baby's health problem, and history of depression.

Abbreviations: CI, confidence interval; ICSI, Intracytoplasmic sperm injection; IUI, Intrauterine insemination; IVF, In–vitro fertilization; OI, Ovulation inducer, RR, Relative risk; SI, Scheduled intercourse.

*p < 0.05, **p<0.01, ***p<0.001. n = 2105

Supplementary Table 4B. The association between the way of getting pregnant and death fantasies within 2 years after delivery

	Men, n = 1236			Women, n = 5635		
	Death fantasies			Death fantasies		
	n = 98			n = 598		
			Model 3			Model 3
	n	Number of cases	RR (95% CI)	n	Number of cases	RR (95% CI)
Expected spontaneous pregnancy	906	64	1	3668	342	1
Unexpected spontaneous pregnancy	126	11	1.01 (0.54–1.91)	1028	160	**1.40 (1.17–1.67)*** **
Fertility treatment (SI/OI)	84	11	1.34 (0.79–2.25)	393	35	0.94 (0.68–1.30)
Fertility treatment (AIH)	41	6	**2.41 (1.13–5.17)***	166	15	0.95 (0.57–1.57)
Fertility treatment (IVF)	79	6	1.13 (0.48–2.69)	380	46	**1.40 (1.04–1.88)***
Aging (10 years increment)			0.96 (0.81–1.14)			**0.91 (0.83–0.99)***
Junior high or high school graduate			0.71 (0.36–1.37)			0.99 (0.81–1.21)
Former smoker (vs. Never smoker)			1.55 (0.99–2.42)			**1.27 (1.07–1.52)****
Current smoker (vs. Never smoker)			1.56 (0.98–2.50)			**1.68 (1.26–2.23)*****
Former drinker (vs. Never drinker)			1.10 (0.58–2.07)			1.13 (0.90–1.42)
Current drinker (vs. Current drinker)			0.80 (0.43–1.46)			1.12 (0.87–1.45)
Equivalized household income Q4 (vs. Q5)			1.14 (0.56–2.31)			0.99 (0.77–1.27)
Equivalized household income Q3 (vs. Q5)			1.53 (0.81–2.89)			0.89 (0.69–1.15)
Equivalized household income Q2 (vs. Q5)			1.64 (0.86–3.13)			1.06 (0.84–1.34)
Equivalized household income Q1 (vs. Q5)			1.49 (0.77–2.85)			**1.25 (1.00–1.57)***
A child living together			0.71 (0.48–1.05)			1.11 (0.94–1.31)

Two or more children living together	–	–
Recurrent pregnancy loss	1.43 (0.73–2.81)	0.92 (0.69–1.23)
Health problem of fetal/infant/child	**2.38 (1.21–4.69)***	**1.39 (1.00–1.94)***
History of depression	**6.65 (4.53–9.78)*****	**3.04 (2.55–3.61)*****
Paternal leave	0.97 (0.66–1.42)	1.12 (0.87–1.45)
Maternal leave	1.21 (0.83–1.75)	**0.69 (0.58–0.82)*****
Low birth weight infant	0.99 (0.46–2.12)	1.09 (0.84–1.40)

Model 3: Adjusted for age, junior high or high school graduate, smoking, drinking, equivalized income, number of children living together, recurrent pregnancy loss, baby's health problem, history of depression. paternal leave, maternal leave of partner, and low birth weight infant.

Abbreviations: CI, confidence interval; ICSI, Intracytoplasmic sperm injection; IUI, Intrauterine insemination; IVF, In–vitro fertilization; OI, Ovulation inducer, RR, Relative risk; SI, Scheduled intercourse.

*p < 0.05, **p<0.01, ***p<0.001. n = 6871
